# Predictive Scoring and Risk Factors of Early Recurrence after Percutaneous Endoscopic Lumbar Discectomy

**DOI:** 10.1155/2019/6492675

**Published:** 2019-11-07

**Authors:** Hyeun Sung Kim, Jong Duck You, Chang Il Ju

**Affiliations:** ^1^Department of Neurosurgery, Nanoori Hospital, Seoul, Republic of Korea; ^2^Department of Neurosurgery, Chosun University Hospital, Gwangju, Republic of Korea

## Abstract

**Purpose:**

To predict the early recurrence after full endoscopic lumbar discectomy, we analyzed factors related to demographic factor anatomical factors, operative method, and postoperative management, and predicted the possibility of recurrence according to the scoring system.

**Materials and Methods:**

In this prospective study, we enrolled 300 patients who underwent 1 out of 3 surgical procedures. The patients were randomized into one of the following groups: group A (*n* = 100), transforaminal inside-out approach; group B (*n* = 100), transforaminal outside-in approach; and group C (*n* = 100), interlaminar approach. The clinical results were evaluated by a visual analogue scale (VAS). Related factors evaluated with points of (A) demographic factors: (1) age, (2) gender, (3) BMI, (B) anatomical factors: (4) disc degeneration scale, (5) modic change, (6) number of involved disc herniation, (7) history of discectomy (first, recurred), (8) herniated disc level, (9) disc height, (10) segmental dynamic motion, (11) disc location, (C) operation factors: (12) annulus preservation along the disc protrusion, (13) approach method (transforaminal inside-out, transforaminal outside-in, interlaminar); (D) postoperative care factors: (14) early ambulation, (15) spinal orthosis (corset) application. Among these, we analyzed statistically significant recurrence risk factors after PELD in all patients and early recurrence predicting score ratio was obtained.

**Results:**

The overall recurrence rate was 9.33%. The recurrence rate was 11%, 10%, and 7% for groups A, B, and C, respectively. Average early recurrence time was 3.26 months. The change in preoperative and postoperative VAS score was from 8.07 to 1.39, 8.34 to 1.34, and 8.14 to 1.86 in groups A, B, and C, respectively. The recurrence rate based on the (1) age was <40 years: 5.22% (6/115), 41–60 years: 16.1% (20/124), and >61 years: 3.07% (2/65); (2) gender was male: 13/139 (9.35%), female: 15/161 (9.32%); (3) BMI was obese: 17.57% (13/74), overweight: 11.6% (9/77), underweight: 6.35% (4/63), and normal weight: 2.33% (2/86); (4) degeneration scale was grades 1–2: 2% (1/50), grade 3: 7.4% (10/135), and grades 4–5: 14.8% (17/115); (5) modic change was type I: 25% (3/12), type II: 14.3% (1/7), type III: 33% (1/3), and no modic change: 8.27% (23/278); (6) number of involved disc herniation was 1 level: 3.9% (5/128), 2 level: 10.4% (13/125), 3 levels: 18.9% (7/37), and 4 levels: 30% (3/10); (7) history of discectomy was first: 8.83% (25/283) and repeated: 17.65% (3/17); (8) herniated disc level was L1–L2/L2–L3/L3–L4: 3.95% (3/76) and L4–L5: 14.6% (18/123); (9) disc height was <80%: 17.14% (6/35), 81%–100%: 8.16% (12/147), and >101%: 8.5% (10/118); (10) segmental dynamic motion was 1–10°: 8.58% (20/233) and 11–20° : 11.9% (8/67); (11) disc location was central: 7.41% (2/27), foraminal: 3.03% (2/66), and inferior/superior/paracentral: 11.59% (24/207); (12) radical annulotomy was 8.05% (7/87) vs. 9.86% (21/213); (13) approach method was transforaminal (inside-out): 11% (11/100), transforaminal (outside-in): 10% (10/100), and interlaminar: 7% (7/100); (14) early ambulation was 16.42% (23/140) vs. 3.13% (5/160); and (15) spinal orthosis application was 7.35% (10/136) vs. 10.98% (18/164). According to the above results, after summation of all scores, the early recurrence predicting score: recurrence rate ratio was 1–4: 0% (0/23), 5–8: 7.1% (13/183), 9–12: 8% (6/75) and 13–16 100% (10/10).

**Conclusions:**

Early recurrence after PELD is associated with several risk factors such as BMI, degeneration scale, combined HNP, and early ambulation. If we use the predicting score, we can postulate the occurrence of early recurrence after PELD. Knowing the predictive factors prior to surgical intervention will allow us to decrease the early recurrence rate after PELD.

## 1. Introduction

Recently, percutaneous endoscopic lumbar discectomy (PELD) has been popularized as an alternative to the traditional open discectomy. Like other surgical techniques, minimally invasive spine surgery is becoming the preferred method for both spinal surgeons and patients undergoing surgery for symptomatic lumbar disc herniation. In general, PELD has been performed by two common working pathways such as the transforaminal and interlaminar approach.

Although good surgical outcomes of PELD have been reported in many literatures for the treatment of various lumbar disc herniations, many surgeons are still experiencing endoscopic operative failure [1–10].

Endoscopic operative failure was defined as: (1) intracanal lower lumbar (L3–L4, L4–L5, and L5–S1) disc herniation that required subsequent surgery because of persistent symptoms within 2 weeks after surgery; (2) no pain-free interval from the first operation to the subsequent procedure; and (3) verification of remnant fragments by radiologic studies [11].

One of the most common complication after PELD is recurrent disc herniation. Recurrent lumbar disc herniation is defined as the recurrence of disc herniation at the same site of a previous discectomy, after an initial period of symptomatic improvement. This represents a significant complication of surgical failure, occurring in approximately 5–11% of discectomies [12–15].

We defined early recurrence as the recurrence of disc herniation within 6 months after PELD with a successful pain-free interval and complete removal of the protruding disc by follow-up MRI. The purpose of this study was to evaluate the risk factors related to early recurrence after PELD.

## 2. Materials and Methods

### 2.1. Materials

#### 2.1.1. Patients

Between May 2012 and November 2017, we retrospectively reviewed 300 patients with lumbar disc herniation and performed PELD. All patients were followed-up for at least 6 months. The exclusion criteria were patients who were lost to follow-up in less than 6 months and those with pathologic degenerative spine disease (e.g., spinal stenosis, spondylolisthesis, and synovial cyst).

The patients included in this study met the following inclusion criteria: (1) transforaminal approach: patients who had undergone a surgical procedure above the L4–L5 level and interlaminar approach: patients who had undergone a surgical procedure at the L5–S1 level, (2) postoperative MRI showed complete removal of the protruded disc, (3) recurred radiculopathic leg pain after successful symptom-free interval at least longer than 2 weeks, (4) follow-up MRI showed newly developed disc protrusion in the previously operated site.

**Figure 1:**
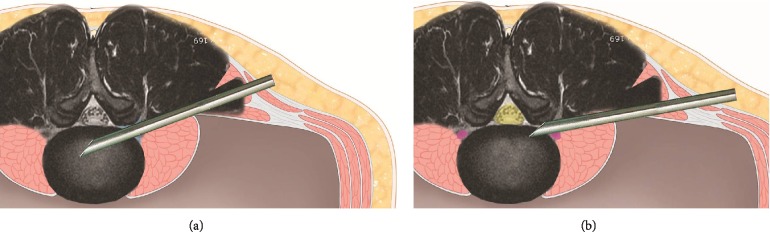
Transforaminal Inside-out and Outside-in technique. The technique of endoscopic transforaminal approach can be divided into the inside-out or outside-in techniques, based on the sequence method of whether the working channel was inserted into the disc space first (a) and then approaches the epidural space (out of disc space) later or in a reverse order (b).

Patients were classified into three categories according to the endoscopic approach as follows: (1) group A: transforaminal inside-out approach, (2) group B: transforaminal outside-in approach, and (3) group C: interlaminar approach (Table 1, Figure 1).

All endoscopic surgeries were performed by an expert surgeon with at least over 5 years and 500 cases of experience in endoscopic surgery. Possible risk factors for early recurrence of lumbar disc herniation were retrospectively evaluated and included the following: Demographic factors (age, sex, and body mass index); Anatomical factors (disc degeneration scale, Modic change, number of disc herniation, history of discectomy, disc location, herniated disc level, disc height, and segmental dynamic motion), operation factors (annulus preservation, transforaminal inside-out vs outside-in vs interlaminar approach) and postoperative care factors (early ambulation, spinal orthosis).

#### 2.1.2. Follow-Up and New Symptomatic Relapsed Disc Herniation

Patients were followed-up regularly at 2 weeks, 1 month, and every 3 months during the first year after the procedure and then on a yearly basis.

#### 2.1.3. Review of Patient Data

Possible risk factors for new symptomatic recurrent disc herniation were retrospectively evaluated and included the following: demographic factors (age, sex, and BMI); disc factor (disc degeneration scale, modic change, number of disc herniation, history of discectomy, disc location, herniated disc level, disc height, and segmental dynamic motion); operation factors (annulus preservation, inside-out/outside-in approach); and postoperative care factors (early ambulation, spinal orthosis).

Based on this data, we developed a predictive scoring system to evaluate the risk of an early recurrent disc herniation.

We attempted to develop a scoring system for predicting recurrent lumbar disc herniation based on the collected data. We analyzed the data of individuals who had previous endoscopic discectomy and those with sufficient information. All radiographic information was extracted from the medical record system including disc degeneration scale, combined disc, herniated disc level, disc height, and segmental dynamic motion in the recurrent herniated disc levels. We obtained all the values of the segmental dynamic motion from the lumbar flexion/extension lateral image before reoperation.

In the evaluation of various parameters, we assigned the following points based on the (1) age (0 point: <40 years, 2 points: 40–60 years, 0 point: <60 years); (2) gender (0 point: male, 0 point: female); (3) BMI (0 point: <25 kg/m^2^, 1 point: 25–30 kg/m^2^, 2 points: >30 kg/m^2^); (4) disc degeneration scale (0 point: grade 1–2, 1 point: grade 3, 2 points: grade 4–5); (5) modic change scale (0 point: no modic change, 1 point: type II or III, 2 points: type I); (6) number of involved disc herniation (0 point: 1 level, 1 point: 2 levels, 2 points: 3 levels, 3 points: 4 levels); (7) history of discectomy (0 point: first, 0 point: more than second); (8) disc location (1 point: central, 0 point: foraminal or far lateral, 2 points: paracentral, 3 points: sequestrated migration); (9) herniated disc level (0 point: L1–L2, L2–L3, or L3–4, 1 points: L4–L5); (10) disc height (2 points: <80%, 1 point: 80–100%, 0 point: >100%); (11) segmental dynamic motion (0 point: groups 1–10, 0 point: groups 11–20); (12) annulus preservation (0 point: minimal annulotomy, 1 point: radical resection); (13) early ambulation (1 points: early ambulation, 0 point: bed rest); (14) spinal orthosis (corset) application (0 point: corset applied, 1 point: no corset).

#### 2.1.4. Early Recurrence of Scoring System after Endoscopic Lumbar Discectomy

**Figure 2:**
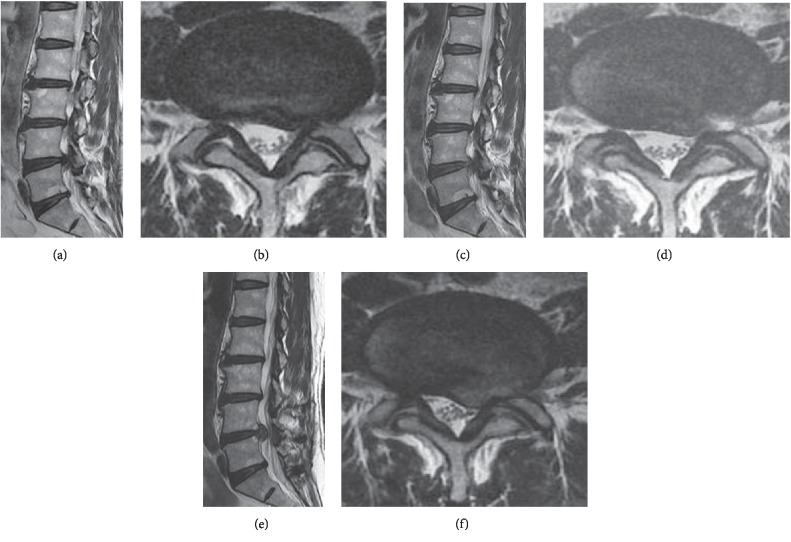
Case of early recurrence after PELD. Preoperative MRI shows L4–5 disc herniation left paracentral and foraminal type (a, b). Immediate postoperative MRI image shows L4–5 left side disc removed and left nerve root decompressed (c, d). However, 4 month later, follow-up MRI shows L4–5 disc reherniation again at same operated site (e, f). According to scoring system, (1) age: 47 (2 point), (2) gender: male (0 point), (3) BMI: 28.3 kg/m^2^ (1 point), (4) disc degeneration scale: 3 scale (1 point), (5) Modic change (0 point), (6) combined HNP: 2 level (1 point), (7) disc herniation episode: first (0 point), (8) annulus preservation: minimal annulotomy (0 point), (9) approach: transforaminal outside-in (0 point) (10) disc location: paracentral (2 points) (11) herniated disc level: L4–5 (1 point), (12) disc height: 80–100% (0 point), (13) segmental dynamic motion: group 5 (0 point), (14) early ambulation: walking within 2 days (1 point), (15) spinal orthosis: no corset (1 point). Total score: 10 points (group C) .

**Figure 3:**
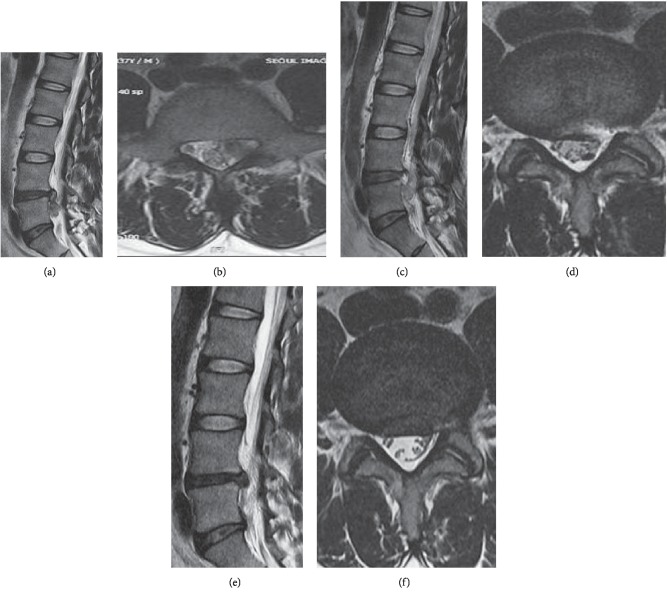
Case of early recurrence after PELD. Preoperative MRI shows L4–5 disc herniation left downward migrated type (a, b). Immediate postoperative MRI image shows L4–5 left side disc removed and left nerve root decompressed (c, d). However, 4 month later, follow-up MRI shows L4–5 disc reherination again at same operated site (e, f). According to scoring system, (1) age: 55 (2 point), (2) gender: female (0 point), (3) BMI: 23 kg/m^2^ (0 point), (4) disc degeneration scale: 3 scale (1 point), (5) Modic change (0 point), (6) combined HNP: 1 level (0 point), (7) disc herniation episode: first (0 point), (8) annulus preservation: minimal annulotomy (0 point), (9) approach: transforaminal outside-in (0 point) (10) disc location: paracentral downward migrated (2 points) (11) herniated disc level: L4–5 (1 point), (12) disc height: 66% (1 point), (13) segmental dynamic motion: group 7 (0 point), (14) early ambulation: bed resting 5 days (0 point), (15) spinal orthosis: corset (0 point). Total score: 7 points (group B).

According to the total summation of points, we classified all the subjects into four groups groups (I, II, III, and IV) and investigated the correlation of risk for early recurrence. Each group and early recurrence rates were comparatively analyzed (Figures 2 and 3).

#### 2.1.5. Statistical Analysis

Age, gender, BMI, disc degeneration scale, Modic change, combined disc, herniated disc level, disc height, segmental dynamic motion in the recurrent herniated disc levels, early ambulation, and spinal orthosis were recorded. Baseline comparisons were performed using the paired *t*-test; chi-squared test, and risk factors for early recurrent disc herniation were analyzed using the logistic regression test. SPSS ver. 15.0 software (SPSS Inc., Chicago, IL, USA) was used for all statistical analyses, and *P*-values < 0.05 were considered statistically significant.

## 3. Results

A total of 300 patients (group A: 100, group B: 100, group C: 100) were enrolled in this study. There were 139 males and 161 females. The mean age was 46.72 ± 15.24 years (range 19–93 years) and the mean follow-up duration was 35.5 months (range, 6–75 months). The average age was 46.51 ± 18.14 years for group A, 45.65 ± 15.08 years for group B and 47.29 ± 14.56 years for group C (Table 2).

The mean follow-up period for each group was 21.12 ± 4.57 months in group A, 12.54 ± 3.41 months in group B, and 19.00 ± 4.42 months in group C. The total early recurrence rate after PELD was 9.33% (28/300), and the recurrence rate in each group was 11% (11/100) for group A, 10% (10/100) for group B, and 7% (7/100) for group C. Overall, the mean recurrence time after disc removal was 3.26 months.

The changes of the visual analogue scale (VAS) score before and after endoscopic surgery improved from 8.18 ± 0.78 preoperatively to 1.55 ± 1.0 postoperatively, 9.07 ± 0.77 to 1.39 ± 2 0.92 in group A, 8.34 ± 0.50 to 1.34 ± 0.93 in group B, and 8.14 ± 0.82 to 1.86 ± 1.09 in group C (Table 2).

### 3.1. Early Recurrence Rates after PELD

Of the 300 patients who were followed-up, early recurrence occurred in 28 cases (9.3%) after PELD.

The recurrence rate after removal of the discs using the transforaminal approach was 10.5% (21/200) for groups A and B and 7% (7/100) for group C using the interlaminar approach. Overall, the mean recurrence time after disc removal was 3.58 months. The early recurrence rate was higher in the group using the transforaminal approach (groups A and B) than in the group using the interlaminar approach (group C); however, there was no difference in the surgical approach method.

### 3.2. Changes in VAS

After PELD, the preoperative pain reduced significantly. Moreover, irrespective of the endoscopic approach used, the postoperative VAS score was reduced significantly in all groups [mean preoperative VAS vs postoperative VAS: group A, 8.07 ± 0.77 vs. 1.39 ± 0.92; group B, 8.34 ± 0.50 vs. 1.34 ± 0.93; and group C, 8.14 ± 0.82 vs. 1.86 ± 1.09. (*P* ≤ 0.05) (Table 2).

#### 3.2.1. Demographic Factors

*(1) Age and Gender.* The early recurrence rate was related to age. Relatively high recurrence rates (20/124, 16.1%) were seen in patients between 40 and 60 years of age. A similar recurrence rate was observed in the groups below 40 years old (6/115, 5.22%) and those over 60 years old (2/65, 3.07%). There was no statistically significant difference in the early relapse rate for age and gender: male (13/139, 9.35%) vs. female (15/161, 9.32%) (*P* < 0.05) (Table 3).

*(2) Body Mass Index (BMI).* The early recurrence rate was related to BMI which is a simple calculation using a person's height and weight. The formula is BMI = kg/m^2^ where kg is a person's weight in kilograms and m^2^ is their height in meters squared. BMI ranges are underweight: <18.5 kg/m^2^, normal weight: 18.5–25 kg/m^2^, overweight: 25–30 kg/m^2^, and obese: >30 kg/m^2^. Relatively high recurrence rates were seen in the obese (13/74, 17.57%) and overweight (9/77, 11.69%) patients. A similar recurrence rate was observed in the underweight (4/63, 6.35%) and normal-weight (2/86, 2.33%) patients (*P* ≤ 0.05) (Table 3).

#### 3.2.2. Anatomical Factors

*(1) Disc Degeneration Scale.* In the present study, according to the grading system of Pfirrmann et al. [16], we classified the disc degeneration scale into the following three scales: 1 scale (mildly degenerated), 2 scale (moderately degenerated), and 3 scale (severely degenerated: completely blackened). The classification by Pfirrmann et al. [16] is useful in assessing the degrees of disc degeneration on T2-weighted images: grade 1 (normal shape, no horizontal bands, clear distinction of the nuclei and annuli), grade 2 (nonhomogeneous shape with horizontal bands, some blurring between the nuclei and annuli), grade 3 (nonhomogeneous shape with blurring between the nuclei and annuli, annuli shape is still recognizable), grade 4 (nonhomogeneous shape with hypointensity, annuli shape is not intact and distinction between the nuclei and annuli is impossible, disc height is usually decreased), and grade 5 (same as grade 4 but with collapsed disc space). Grades 1 to 2 were classified as normal discs, while grades 3 to 5 were defined as degenerative.

Early disc recurrence showed a good relation with the disc degeneration scale; the greater the disc degeneration scale, the more frequently disc herniation recurred. Two percent (1 out of 50 cases) of early recurrent disc herniation occurred in patients with grades 1 to 2 disc degeneration. Meanwhile, 7.4% (10 of 135 cases) and 14.8% (17 of 115 cases) in the disc degeneration of 3 grade and 4–5 grade (*P* ≤ 0.05) (Table 3).

*(2) Modic Change.* The early disc recurrence rate increased in Modic change. Modic changes are pathological changes in the bones of the spine and the vertebrae. These changes are situated both in the vertebral body and in the end plate of the neighboring disc. In Modic type I, there is vascular development in the vertebral body, with findings of inflammation and edema, but no trabecular damage or marrow changes. In Modic type II, there are changes in the bone marrow, with fatty replacement of formerly red, cellular marrow normally seen there. In Modic type II, the marrow is substituted by the visceral fat, the same kind of fat we have on our hips and bellies. Modic type III changes are less common, with fractures of the trabecular bone, along with trabecular shortening and widening.

In our study, there are 22% (5/22 cases) of early recurrence rate in the Modic change group; 25% (3/12 cases), 14.3% (1/7 cases), and 33% (1/3 cases) showed type I, II, and III Modic change, respectively; however, only 8.27% (23/278 cases) showed disc recurrence for no Modic change group. (*P* > 0.05) (Table 3).

*(3) Number of Involved Disc Herniation.* The early disc relapse rate increased in proportion to the number of involved disc herniation levels. About 10.4% (13/125 cases), 18.9% (7/37 cases), and 30% (3/10 cases) showed early relapse in 2, 3, and 4 levels of involved disc herniation cases, respectively; however, only 3.9% (5/128 cases) showed disc relapse for one involved level disc herniation. (*P* ≤ 0.05) (Table 3).

*(4) History of Surgery for Disc Herniation.* The early recurrence rate was 8.83% (25/283) in patients who underwent endoscopic discectomy for the first time after being diagnosed with herniated disc and 17.65% (3/17) in patients who underwent endoscopic reoperation after the past surgery. There was no statistically significant difference between the two groups (*P* > 0.05) (Table 3).

*(5) Location of Disc Herniation.* The recurrence rate after PELD according to the type of disc location was commonly found in the paracentral type of disc herniation followed by the central and far lateral types. In particular, 11.59% (24/207 cases) showed early recurrence in the paracentral type (including superior or inferior migration type) of disc herniation. However, only 7.41% (2/27 cases) and 3.03% (2/66 cases) showed early recurrence in the central type and foraminal type (including extraforaminal type) disc herniation, respectively (*P* > 0.05) (Table 3).

*(6) Level of Disc Herniation.* The rate of recurrence was significantly higher in L4–L5 than in the upper lumbar disc herniation. Early recurrence rate was 14.6% (18/123) in cases of L4–L5 disc herniationn, 7.0% (7/100) in L5–S1 and 3.95% (3/76) in cases of upper lumbar disc herniation (L1–L2, L2–L3, L3–L4) (*P* > 0.05) (Table 3).

*(7) Disc Height.* Early disc relapse showed good relation with the disc height; the smaller the disc height, the more frequently disc herniation recurred. About 17.14% (6 out of 35 cases) of early recurrence occurred in the cases with less than 80% of normal disc height. Meanwhile, 8.16% (12 out of 147 cases) and 8.5% (10 out of 118 cases) of early recurrence occurred in the cases with 80–100% of normal disc height and in the cases with larger than normal disc height (*P* > 0.05) (Table 3).

*(8) Segmental Dynamic Motion.* Early recurrence rate was 8.58% (20/233) in group between 1 and 10 of segmental dynamic motion and 11.9% (8/67) in group between 11 and 20 of segmental dynamic motion. There was no statistically significant difference between the two groups (*P* > 0.05) (Table 3).

#### 3.2.3. Operation Factors

*(1) Approaching Method.* The total early recurrence rate after PELD was 9.33% (28/300), and the recurrence rate in each group was 11% (11/100) for group A, 10% (10/100) for group B, and 7% (7/100) for group C. The recurrence rate of the group using the transforaminal approach was 12% (21/200) for groups A and B and that using the interlaminar approach was 7% (7/100) for group C. The early recurrence rate was higher in the group using the transforaminal approach (groups A and B) than in the group using the interlaminar approach (group C); however, there was no significant difference in the surgical approach method (Table 3).

*(2) Annulus Preservation.* The early recurrence rate was 8.05% (7/87) in cases of endoscopic discectomy preserving the annulus without radical annulotomy and 9.86% (21/213) in cases of endoscopic discectomy with radical annulotomy. There was no statistically significant difference between the two groups (*P* > 0.05) (Table 3).

#### 3.2.4. Postoperative Care Factor

*(1) Early Ambulation.* Early ambulation is a technique in the postoperative care in which a patient gets out of bed and engages in light activity (such as sitting, standing, or walking) as soon as possible after an operation. Early ambulation was possible after 1 day.

Early recurrence rate was 16.42% (23/140) in the early ambulation group and 3.13% (5/160) in the group with bed rest longer than 3 days after surgery. (*P* ≤ 0.005) (Table 3).

*(2) Orthosis Application.* Corsets were used to wear orthoses, and they were worn immediately after surgery and were compared with nonwearing groups. Early recurrence rate was 7.35% (10/136) in the corset group and 10.98% (18/164) in the noncorset group (*P* > 0.05) (Table 3).

### 3.3. Early Recurrence Rate according to the Scoring System

Based on the factors related to early recurrences including the age, gender, disc degeneration, combined disc herniation, disc herniation history, disc location (central, foraminal or far lateral, paracentral, and sequestrated migration), annulus preservation, herniated disc level, disc height, and segmental dynamic motion, we developed the scoring system and applied it to all cases of early recurrence. We classified all cases into four groups (I, II, III, IV) according to the early recurrence score. Groups I, II, III, and IV were defined by total scores of 0–4, 4–8, 9–12, and 13–16, respectively.

According to early recurrence score, groups I, II, III, and IV showed an early recurrence rate of 0% (0/32 cases), 7.1% (13/183 cases), 8.0% (6/75 cases), and 100% (10/10 cases) (Figures 2 and 3).

Therefore, the total score had a close relation with the risk of early recurrence of disc herniation after endoscopic lumbar discectomy. Groups I, II, III, and IV could be classified as low risk, mild~ moderate risk, high risk groups, respectively (*P* ≤ 0.05) (Table 4).

## 4. Discussion

Recurrent lumbar disc herniation is defined as a recurrence of disc herniation at the same site of a previous discectomy in a patient who has experienced a pain-free interval after surgery. However, the minimum length of the pain-free interval is debatable, ranging from any interval of pain resolution to 6 months [15, 17].

Moreover, recurrent disc herniation should be discriminated from incomplete discectomy or endoscopic operative failure.

Lee et al. [11] reported endoscopic operative failure as: (1) intracanal lower lumbar (L3–L4, L4–L5, and L5–S1) disc herniation that required subsequent surgery because of persistent symptoms within the 2 weeks after surgery; (2) no pain-free interval from the first operation to the subsequent procedure; and (3) verification of remnant fragments by radiologic studies.

We defined the early recurrence of disc herniation after PELD as a recurrence of disc herniation within 6 months after at least 2 weeks of successful pain-free interval with complete removal of the protruding disc by follow-up MRI.

Several studies reported that the recurrent disc herniation represents a significant cause of surgical failure, occurring in approximately 5–11% of discectomies [12–15]. The recurrence rate after PELD has been reported to be 0%–7.4% [17–20]. Some researchers showed that there was no significant difference in the recurrence rate between open surgery and PELD [21, 22].

Kim et al. [23] reported old age, high BMI, protrusion type of disc herniation, and positive Modic changes as risk factors after percutaneous endoscopic discectomy.

Swartz and Trost [24], however, found that age, gender, smoking status, level of herniation, and duration of symptoms were not associated with RLDH.

Yao et al. [25] reported that obesity (BMI ≥25 kg/m^2^) was the most robust risk factor responsible for recurrence after PELD. Also, they insisted that older age (≥50 years old), learning curve of the surgeon (<200 cases), treatment period (March 2005 to September 2010), and central location of herniation were closely associated with recurrent herniation after successful PELD.

In our department, the early recurrence rate after successful PELD between March 2005 and March 2016 was 9.5%. Revision surgery is necessary for patients who fail to respond to conservative therapy. To explore independent risk factors for early relapse after PELD, data from 300 patients with after PELD were analyzed, and life factor (age, sex); disc factor (disc degeneration scale, combined disc, disc herniation event); operation factor (disc location, annulus preservation, and inside-out/outside-in approach); and segmental stability factor (herniated disc level, disc height, and segmental dynamic motion).

Unlike other reports, in our study, early recurrence rate was relatively high in the middle age groups (40–60 years) than in young and old age groups. The reason for the high early recurrence rate in the middle age group is that physical activity is similar to that of the younger age group; however, there is more degenerative disc change in the young age group. On the other hand, physical activity is higher than that of old age group with similar degenerative disc change. Also, another reason for the high recurrence rate in the age group of 40 ~ 60s is that the stenosis increases rapidly in the 60s, however, in this study, the spinal stenosis is excluded.

The previous clinical studies indicated that an age of more than 40 years was a predisposing factor to failure of the operation [3]. Older discs generally have a greater degree of degenerative changes, and the remaining discs after discectomy are more susceptible to mechanical damage due to physical load on the incision site. The disc degeneration grade proposed by Pfirrmann et al. [16] was statistically significant in the recurrent group in contrast to the nonrecurrent group; the greater the disc degeneration scale, the more frequently disc herniation recurred. These findings provide evidence that the healing processes that occur in the outer lamellae after annular injury may not be sufficient for effective reconstitution of the external annulus in degenerated discs [26, 27].

The result of this study showed that patients with combined multi-level disc herniation were more likely to experience recurrent disc herniation compared to patients with single-level disc herniation. It is reasonable that multi-level intervertebral disc herniation typically has a higher disc degeneration, and the remaining intervertebral disc damaged during surgery can easily prolapse in response to mechanical overload.

However, the number of previous discectomies is not related to the early relapse of disc herniation. If discectomy is successful, the number of previous operations will not increase the recurrence rate.

The results of this study showed that patients with paracentral disc herniation were more likely to experience early relapse compared to patients with central and far lateral herniation. Yao et al. [25] reported that patients with central herniation were more likely to experience recurrent herniation compared to patients with paramedian herniation. They believed that the role of this risk factor is highly related to the choice of the working channel position. The key point of PELD is to place the working channel near the herniated content. For the treatment of central herniation, the working channel is placed inside the nucleus pulposus with a very steep trajectory angle. As a result, the ruptured intervertebral disc is not easily accessible. However, this is contradictory to our opinion. For the central disc herniation, the working channel should be placed inside the nucleus pulposus with a more horizontal trajectory angle. Using this approach we could remove more centrally located disc herniation aggressively. However, in the cases of paracentral and far lateral disc herniation, approaching trajectory should be more vertical.

We believe that this difference in approaching trajectory makes the range and amount of discs that can be removed different, and the remnant disc material would be an important role of recurrence after PELD.

The degree of removal of the annulus fibrosus during discectomy may vary from person to person. In our study, the method of extracting the nucleus by putting the forceps through only the small hole of the annulus did not reduce the early relapse rate compared to the removal of the annulus fibrosus. Perhaps, the smaller the hole in the annulus, the higher the pressure in the disc space would be. Hence, there seems to be no difference between the two groups.

The technique of endoscopic transforaminal approach can be divided into the inside-out or outside-in techniques, based on the sequence method of whether the working channel was inserted into the disc space first and then approaches the epidural space (out of disc space) later or in a reverse order.

The inside-out technique is a method of removing the herniated disc by inserting the working sheath into the disc space and performing the discectomy, which is advantageous for the beginner. In contrast, the outside-in technique start from docking the working sheath in the extradiscal space of the safety zone and then approaching to the epidural space. This technique is advantageous method for aminoplasty to remove the migrated disc herniation in narrowed safety zones.

However, there is no difference in the recurrence rate between the two groups. In fact, many of the experienced surgeons were able to change two methods according to the surgical situation, and there was no difference in the results.

L4–L5 is the most common early recurrence site because it is the most weight loaded level. However, the number of cases of PELD was overwhelming at L4–L5. Therefore, it is believed that there is a statistical limit to compare with recurrence rates of other levels.

Our study showed that preoperative intervertebral disc heights were statistically not significant in early recurrent disc herniation (*P* = 0.255). However, disc collapsed height was less than 80% showed twice recurrent rate. Especially disc collapsed height was less than 80% showed twice recurrent rate. Axelsson et al. [28] reported that degenerative segments with preserved disc height have a latent instability compared to segments with collapsed discs. Hasegawa et al. [29] reported that the restabilization stage begins when the disc height is reduced by 50%.

Early ambulation and orthosis application may affect the recurrence rate of lumbar intervertebral disc herniation after endoscopic discectomy. This suggests that the body weight is repeatedly applied to the remaining nucleus in the partially removed disc space. This will increase the risk of recurrence of the disc herniation by increasing the disc pressure. It is believed that wearing corset to disperse body weight will reduce the load on the nucleus pulposus and lower the pressure in the intervertebral disc to prevent recurrent disc herniation. However, corset wearing has limitation to prevent early recurrent disc herniation.

## 5. Conclusion

The early recurrent disc herniation after PELD is defined as recurrence of disc herniation within 6 months after successful pain-free interval for at least 2 weeks and complete removal of the protruding disc by follow-up MRI. It is associated with several factors such as BMI, degeneration scale, combined HNP, and early ambulation. Except for the operation factor and segmental instability factor, Life factor and postoperative factor affect the recurrence. That is, the operation factor has no significant effect on recurrence.

It may play an important role in the failure of endoscopic surgery. According to our scoring system, the total score was associated to the risk of early recurrence of disc herniation after endoscopic lumbar discectomy. If the score is high, the patients have a greater chance of early recurrence. Therefore, more attention must be provided to such patients; indeed, providing education with respect to strict bed rest, spinal bracing. Knowing the predictive factors prior to surgical intervention will allow us to decrease the early relapse rate after PELD.

## Figures and Tables

**Table 1:** Classification of group of percutaneous endoscopic lumbar discectomy according to approach method.

Group	Number	Approach
Group A	100	Transforaminal (Inside-out)
Group B	100	Transforaminal (Outside-in)
Group C	100	Interlaminar

**Table 2:** Surgical Outcome and Recurrence Rate according to the endoscopic approaching method.

Group	Follow-up (months)	Mean age	Recurrence	Pre-OP VAS	Post-OP VAS
Group A	21.12 ± 4.57	46.51 ± 18.14	11% (11/100)	8.07 ± 0.77	1.39 ± 0.92
Group B	12.54 ± 3.41	45.65 ± 15.08	10% (10/100)	8.34 ± 0.50	1.34 ± 0.93
Group C	19.0 ± 4.42	47.29 ± 14.56	7% (7/100)	8.14 ± 0.82	1.86 ± 1.09

**Table 3:** Early recurrence rate according to factors after Percutaneous endoscopic lumbar discectomy.

	Factors	Group	Recurrence rate	Score	Relation	(*P* = )
Demographic factors

	Age	~40	6/115 (5.22%)	0	No	0.824
		41~60	20/124 (16.1%)	2		
		61~	2/65 (3.07%)	0		
		Total	28/300 (9.3%)			
	Gender	Male	13/139 (9.35%)	0	No	0.956
		Female	15/161 (9.32%)	0		
		Total	28/300 (9.3%)			
	BMI (kg/m2)	<18.5 kg/m2	4/63 (6.35%)	0	Yes	0.045
		18.5~25	2/86 (2.33%)	0		
		25~30	9/77 (11.69%)	1		
>30		13/74 (17.57%)	2			
Total		28/300 (9.3%)				

Anatomical factors	Disc degeneration scale	Grade 1–2 (mild)	1/50 (2%)	0	Yes	0.018
		3 scale (moderate)	10/135 (7.4%)	1		
		4–5 (severe)	17/115 (14.8%)	2		
		Total	28/300 (9.3%)			
	Modic change	Type I	3/12 (25%)	0	No	0.153
		Type II	1/7 (14.3%)	0		
		Type III	1/3 (33%)	0		
		Total	5/22 (22%)			
	Number of involved disc herniation	One level	5/128 (3.9%)	0	Yes	0.001
		Two level	13/125 (10.4%)	1		
		Three level	7/37 (18.9%)	2		
		Four level	3/10 (30%)	3		
		Total	28/300 (9.3%)			
	History of discectomy	First	25/283 (8.83%)	0	No	0.236
		Reoperation	3/17 (17.65%)	1		
	Location of disc herniation	Paracentral (including sequestrated disc)	24/207 (11.59%)	2	No	0.306
		Central	2/27 (7.41%)	1		
		Foraminal and extraforaminal	2/66 (3.03%)	0		
		Total	28/300 (9.3%)			
	Level of disc herniation	Upper disc (L1–2, L2–3, L3–4)	3/76 (3.95%)	0	Yes	0.174
		L4–5	18/123 (14.6%)	2		
		L5–S1	7/100 (7%)	1		
		Total	28/300 (9.3%)	Total		
	Disc height	Less than 80%	6/35 (17.14%)	1	No	0255
		80%–100%	12/147 (8.16%)	0		
		Larger	10/118 (8.5%)	0		
		Total	28/300 (9.3%)			
	Segmental dynamic motion	Group 1~10	20/233 (8.58%)	0	No	0.558

Operation factors	Approach method	Transforaminal (Inside-out)	11/100 (11%)	0	No	
		Transforaminal (Outside-in)	10/100 (10%)	0		
		Interlaminar	7/100 (7%)	0		
	Annlus preservation	Radical annulotomy	21/213 (9.86%)	0	No	0.625
		Minimal annulotomy	7/87 (8.05%)			
		Total	28/300 (9.3%)			
		Group 11~20	8/67 (11.9%)	0		

Postoperative factors	Early ambulation	Walking within 2 days	23/140 (16.42%)	1	Yes	0.001
		Bed rest longer than 3 days	5/160 (3.13%)	0		
	Orthosis application	Corset apply	10/136 (7.35%)	0	Yes	0.286
		No corset	18/164 (10.98%)	1		

Early recurrence rate according to the scoring system					Yes	0.001

**Table 4:** Early recurrence rate according to groups of predictive recurrence score.

Group	Total score	Early recurrence rate	Risk of early recurrence
Group I	0~4	0% (0/32)	Low
Group II	4~8	7.1% (13/183)	Mild~moderate
Group III	8~12	8.0% (6/75)	Mild~moderate
Group IV	12~16	100% (10/10)	High

## Data Availability

The data used to support the findings of this study are available from the corresponding author upon request.
